# Oncolytic Adenovirus for the Targeting of Paclitaxel-Resistant Breast Cancer Stem Cells

**DOI:** 10.3390/v16040567

**Published:** 2024-04-05

**Authors:** Sacha Robert, Natasha Ivelisse Roman Ortiz, Christopher J. LaRocca, Julie Hanson Ostrander, Julia Davydova

**Affiliations:** 1 Department of Surgery, University of Minnesota, Minneapolis, MN 55455, USA; clarocca@umn.edu; 2 Department of Pharmacology, University of Minnesota, Minneapolis, MN 55455, USA; roman449@umn.edu; 3 Masonic Cancer Center, University of Minnesota, Minneapolis, MN 55455, USA; hans1354@umn.edu; 4 Division of Hematology, Oncology, and Transplantation, Department of Medicine, University of Minnesota, Minneapolis, MN 55455, USA; 5 Institute of Molecular Virology, University of Minnesota, Minneapolis, MN 55455, USA

**Keywords:** human adenovirus, breast cancer, breast cancer stem cell, chemoresistance, oncolytic vectors, sodium iodide symporter

## Abstract

Adjuvant systemic therapies effectively reduce the risk of breast cancer recurrence and metastasis, but therapy resistance can develop in some patients due to breast cancer stem cells (BCSCs). Oncolytic adenovirus (OAd) represents a promising therapeutic approach as it can specifically target cancer cells. However, its potential to target BCSCs remains unclear. Here, we evaluated a Cox-2 promoter-controlled, Ad5/3 fiber-modified OAd designed to encode the human sodium iodide symporter (hNIS) in breast cancer models. To confirm the potential of OAds to target BCSCs, we employed BCSC-enriched estrogen receptor-positive (ER+) paclitaxel-resistant (TaxR) cells and tumorsphere assays. OAd-hNIS demonstrated significantly enhanced binding and superior oncolysis in breast cancer cells, including ER+ cells, while exhibiting no activity in normal mammary epithelial cells. We observed improved NIS expression as the result of adenovirus death protein deletion. OAd-hNIS demonstrated efficacy in targeting TaxR BCSCs, exhibiting superior killing and hNIS expression compared to the parental cells. Our vector was capable of inhibiting tumorsphere formation upon early infection and reversing paclitaxel resistance in TaxR cells. Importantly, OAd-hNIS also destroyed already formed tumorspheres seven days after their initiation. Overall, our findings highlight the promise of OAd-hNIS as a potential tool for studying and targeting ER+ breast cancer recurrence and metastasis.

## 1. Introduction

Breast cancer is today the most common cancer diagnosed in women worldwide, replacing lung cancer, with an average risk of 13% for a woman in the United States to develop breast cancer sometime in her life [1]. The most aggressive form of breast cancer types are known as triple-negative, which lack expression of hormone receptors, including estrogen receptor (ER) and/or progesterone receptor (PR) as well as the expression of the human epidermal growth factor receptor 2 (HER2). Other breast cancer types involve the overexpression of HER2 (HER2+). However, those breast cancer types represent only a fifth of all breast cancer diseases. About 80% of breast cancers are hormone receptor-positive [2]. Thus, several endocrine-targeted therapies have been developed to interfere with ER signaling, block estrogen synthesis, or promote estrogen degradation. These include selective ER modulators such as tamoxifen, aromatase inhibitors like anastrozole and letrozole, and selective ER degraders such as fulvestrant, all of which have proven to be effective in treating ER+ breast cancer [3]. However, metastatic breast cancer that recurs due to de novo or acquired resistance to endocrine and chemotherapy treatments continues to pose a significant health challenge for women with luminal ER+ breast cancer [4]. This critical situation is estimated to impact approximately 10–60% of breast cancer patients in the US [5,6]. ER+ tumor cells that have spread throughout the body can remain dormant for years to decades while remaining viable [7]. A crucial factor contributing to metastasis includes the maintenance and expansion of breast cancer stem cells (BCSCs) [8]. Since BCSCs exhibit enhanced resistance to chemo-, radio-, and endocrine therapies, more efficient treatment options are needed. Adjuvant systemic therapies effectively reduce the risk of local recurrence and the development of distant metastatic disease by treating preexisting, clinically undetectable micrometastatic deposits [4,9,10]. This allows for the development of more reliable therapies to prevent ER+ breast cancer relapses arising from chemo- and endocrine therapy-resistant BCSCs.

One of the major strengths of oncolytic adenoviruses (OAds) is their ability to selectively target cancer cells while sparing healthy cells [11]. This specificity makes them potentially less toxic than traditional chemotherapy and radiation treatments, which can damage both cancerous and healthy cells. Additionally, OAds can replicate within the tumor, leading to the destruction of cancer cells and the release of more virus particles to infect neighboring cancer cells [12]. They may also stimulate the immune system to recognize and attack cancer cells [13]. Furthermore, as a genetically engineered therapy, OAds can be modified and optimized to improve their targeting, replication, and immune-stimulating properties [14]. These advantages make OAd a promising avenue for cancer therapy, particularly for types of cancer that are difficult to treat with traditional methods.

OAd holds immense promise as a therapeutic option for breast cancer, particularly in cases of metastatic disease. Their potential for strategic modifications to improve tumor selectivity positions them as a powerful tool in the fight against breast cancer [15]. Our laboratory engineered and studied OAds encoding the human sodium/iodide symporter (hNIS), a transmembrane protein that can transport iodide ions across cell membranes [16]. Since one of the strengths of OAd-based vectors is their ability to deliver transgenes specifically to cancer cells while sparing healthy cells, it is possible to make these cells take up radioactive iodine, which can then be used for imaging or radiotherapy. NIS-based virotherapy and imaging is a strategy that has been successfully employed by others using different viral constructs and in different cancer models such as prostate [17] and pancreatic cancers [18].

hNIS-expressing OAds created in our laboratory are based on the adenovirus type 5 (Ad5) structure with the NIS gene inserted into the E3 region to allow continual hNIS gene expression as the virus replicates. The virus replication is controlled by the tissue-specific Cox-2 promoter, which has been reported to be expressed in approximatively 36% of human breast cancers and is associated with negative prognosis [19]. To overcome the low expression of the Ad5 binding receptor coxsackievirus and adenovirus receptor (CAR) by cancer cells and improve infectivity, we equipped the virus with an Ad5/Ad3-modified fiber, which allows for CD46 and desmoglein 2 (DSG2) receptor attachment [20,21]. Importantly, CD46 and DGS2 are overexpressed in cancer tissues, including breast cancer [22,23].

A major challenge in treating breast cancer relapses is targeting and killing BCSCs, which share many similarities with normal stem cells, such as the ability to differentiate into multiple cell types, self-renew, proliferate, and maintain neoplastic clonality [24]. Identifying BCSCs involves using an assortment of markers, including cell-surface, nuclear, or cytoplasmic proteins; transcription factors; enzymes; and/or functional attributes, notably the ability to grow as spheroids (referred to as tumorspheres) in ultra-low-attachment plates in serum-free media [25]. However, the potential of OAd to target the breast cancer stem cell population has not been thoroughly studied.

In this study, we sought to investigate whether an OAd designed in our lab to express the hNIS could target breast cancer, including BCSC populations, in a preclinical in vitro setting. For this purpose, we used a new and very relevant model of ER+ paclitaxel-resistant breast cancer, which originates from the MCF-7 cell line (TaxR) [26]. These cells have been shown to be enriched with BCSC markers (e.g., CD44^hi^/CD24^lo^) and display chemoresistance to paclitaxel. Furthermore, in 3D tumorsphere cultures, TaxR generate a high frequency of tumorspheres from the expansion of the subset of BCSCs [27].

Therefore, we believe that this study, by providing a novel approach, unequivocally substantiates the potential of OAd-based virotherapy to deliver a formidable treatment effect in therapy-resistant subsets of advanced breast cancers cells, particularly in targeting BCSCs.

## 2. Materials and Methods

### 2.1. Cell Lines

The human breast cancer cell lines MCF-7, BT-474, AU565, MDA-MB-231, and MDA-MB-468; the lung carcinoma A549 cell line; and the normal human mammary epithelial cell line MCF-12A were obtained from the American Type Culture Collection (Manassas, VA, USA). MCF-7 and BT-474 cells are classified as luminal breast cancer cell lines expressing hormonal (estrogen and progesterone) receptors. The AU565 cell line is from the HER2+ classification. MDA-MB-468 and MDA-MB-231 cells are triple-negative cell lines, not expressing either hormonal receptors or HER2. A549, MDA-MB-231, and MDA-MB-468 were grown in DMEM (Corning, New York, NY, USA) supplemented with 1% penicillin streptomycin (Thermo Fisher Scientific, Waltham, MA, USA), 2 mM L-glutamine (Thermo Fisher Scientific, Waltham, MA, USA), and 5% fetal bovine serum (FBS) (Cytiva-HyClone, Logan, UT, USA). AU565 and BT-474 were grown in RPMI-1640 (Corning, New York, NY, USA) supplemented with 1% penicillin streptomycin (Thermo Fisher Scientific, Waltham, MA, USA), 2 mM L-glutamine (Thermo Fisher Scientific, Waltham, MA, USA), and 5% FBS (Cytiva-HyClone, Logan, UT) for AU565 and 10% FBS (Cytiva-HyClone, Logan, UT, USA) with 10 µg/mL insulin (Thermo Fisher Scientific, Waltham, MA, USA) for BT-474. MCF-7 were grown in EMEM (Corning, New York, NY, USA) supplemented with 1% penicillin streptomycin (Thermo Fisher Scientific, Waltham, MA, USA), 2 mM L-glutamine (Thermo Fisher Scientific, Waltham, MA, USA), 10% FBS (Cytiva-HyClone, Logan, UT, USA), and 0.01 mg/mL insulin (Thermo Fisher Scientific, Waltham, MA, USA). MCF-12A were grown in DMEM/F-12 supplemented with 1% penicillin streptomycin (Thermo Fisher Scientific, Waltham, MA, USA), 2 mM L-glutamine (Thermo Fisher Scientific, Waltham, MA, USA), 5% horse serum (Cytiva-HyClone, Logan, UT, USA), 20 ng/mL recombinant human epithelial growth factor (EGF) (Sigma-Aldrich, St. Louis, MO, USA), 0.5 µg/mL hydrocortisone (Sigma-Aldrich, St. Louis, MO, USA), 0.01 mg/mL insulin (Thermo Fisher Scientific, Waltham, MA, USA), and 0.1 µg/mL cholera toxin (Sigma-Aldrich, St. Louis, MO, USA). The paclitaxel-resistant (TaxR) and parental MCF-7 cell lines were provided kindly by Dr. Julie Ostrander’s laboratory, which developed and characterized the cells [26,27]. Both cell lines were grown in MEM-alpha media (Thermo Fisher Scientific, Waltham, MA, USA) supplemented with 6% FBS (Cytiva-HyClone, Logan, UT, USA), 12 mM HEPES (Thermo Fisher Scientific, Waltham, MA, USA), 1 mM sodium pyruvate (Thermo Fisher Scientific, Waltham, MA, USA), nonessential amino acids (Thermo Fisher Scientific, Waltham, MA, USA), 1 µg/mL insulin (Thermo Fisher Scientific, Waltham, MA, USA), 1 µg/mL hydrocortisone (Sigma-Aldrich, St. Louis, MO, USA), and 12.5 ng/mL EGF (Sigma-Aldrich, St. Louis, MO, USA). TaxR cells are cultured with 2 mM paclitaxel (Invitrogen, Waltham, MA, USA) for both maintenance and 2D culture experiments that include them.

### 2.2. Adenoviral Vectors

The hNIS-expressing OAd vectors were constructed as we described previously [18] (Figure S1). Briefly, all of them are based on the adenovirus type 5 (Ad5) genome. Nonessential E3 genes (6.7 K, gp19 K, RID-α, and -β, 14.7 K) were deleted and replaced with the hNIS gene, while the ADP gene was either conserved (ADP(+)) or deleted (ADP(−)) from the E3 region. The WT replication vector was controlled by the insertion of a tissue-specific Cox-2 promoter into the E1 region to restrict replication and gene expression to permissive tumors. The vectors are equipped with the Ad5/Ad3-modified fiber to overcome CAR deficiency and shift binding to cells expressing CD46 and DSG2 receptors, which were shown overexpressed in breast cancer cells [22,23]. The viruses were propagated in A549 cells, purified by cesium chloride gradient ultracentrifugation, and dialyzed in phosphate-buffered saline (PBS) (Corning, New York, NY, USA) with 10% glycerol (Invitrogen, Waltham, MA, USA). Titration was performed with a pfu assay and optical density-based measurements. The viral particles (vp)/plaque forming units (pfu) ratios for these vectors were in the range of 10–110. Purified virions were confirmed by qPCR to contain the Cox-2 promoter, 5/3 fiber, hNIS, and ADP.

### 2.3. Binding Assay

Cells were plated into 24-well plates and the next day infected with virus at 1 or 10 pfu/cell in 200 µL of appropriate cold media. The cells were immediately incubated in a cold room (4 °C) for 2 h, allowing viruses to bind but preventing internalization of the virus into the cells. Cells were gently washed with PBS (Corning, New York, NY, USA) to remove unbound viruses. Then 200 µL of PBS (Corning, New York, NY, USA) with 20 µL of proteinase K was added to each well, and lysis was performed using lysis buffer from a QIAamp DNA Blood Mini Kit (QIAGEN, Hilden, Germany). Using the manufacturer recommendation, the same kit was used to extract DNA, which was eluted and quantified using a Nanodrop Lite spectrophotometer (Thermo Fisher Scientific, Waltham, MA, USA). qPCR was then performed using E4 primers (Forward 5′-GGAGTGCGCCGAGACAAC-3′, Reverse 3′-ACTACGTCCGGCGTTCCAT-5′) and TaqMan (Probe 5′-G-FAM-TGGCATGACACTACGACCAACACGATCT-TAMRA-3′) on a LightCycler^®^ 480 II (Roche, Basel, Switzerland). Copies of E4 were normalized to 50 ng of total DNA.

### 2.4. Crystal Violet Assay

For MCF-7, AU565, MDA-MB-231, and MDA-MB-468, 1 × 105 cells per well (5 × 10^4^ for A549, 4 × 10^5^ for BT-474, and 6 × 10^4^ for MCF-12A) were plated in 24-well plates and infected at 0.1–10 pfu/cell in 500 μL of appropriate medium. Then, 2 h postinfection, medium was replaced by 1 mL fresh medium and incubated up to 7 days. Medium was aspirated and cells fixed with 10% buffered formalin followed (VWR, Radnor, PA, USA) with 1% crystal violet (Sigma-Aldrich, St. Louis, MO, USA) staining. After staining was completed, the plates were scanned at high resolution using a perfection V33 scanner (Epson, Suwa, Japan). Images were converted to 8-bit on ImageJ version 1.53 t (http:imagej.nih.gov/ij, accessed on 3 January 2023), and the same area selection was applied to each well to measure the mean intensity. The maximal survivability was set for the average of each control untreated well, at each time point, and compared to other treated conditions to determine the percentage of remaining viability.

### 2.5. Immunofluorescent Analyses

(1) Cell line preparation: Cells were grown in 96-well plates (10,000–20,000 cells per well), treated with 0.001–0.5 pfu/cell in 200 μL of growth medium, and incubated for up to 5 days. Cells were washed in PBS (Corning, New York, NY, USA), fixed (4% paraformaldehyde (VWR, Radnor, PA, USA); 20 min; ice), and permeabilized (0.2% Triton X-100 (Sigma-Aldrich, St. Louis, MO, USA); 1 h; room temperature).
(2) Immune staining: Fixed and permeabilized cells were blocked for 2 h at room temperature in a 5% nonfat dry milk (Bio-Rad, Hercules, CA, USA) and 0.2% Triton X-100 solution (Sigma-Aldrich, St. Louis, MO, USA). Cells were washed with PBS (Corning, New York, NY, USA) and incubated at 4 °C overnight with a 5% bovine serum albumin (Roche, Basel, Switzerland), 1% glycine (Sigma-Aldrich, St. Louis, MO, USA), 2% goat serum (MP Biomedicals, Santa Ana, CA, USA), and 0.1% Triton X-100 solution (Sigma-Aldrich, St. Louis, MO, USA) containing the primary antibody anti-NIS (mouse anti-FP5A, MA5-12308, 1:500, Invitrogen, Waltham, MA, USA) and/or anti-CD44 (rabbit anti-CD44, ab189524, 1:200, Abcam, Cambridge, United Kingdom). Cells were then washed with PBS (Corning, New York, NY, USA) and incubated at room temperature, protected from light, for 2 h in the same primary antibody solution, containing either goat anti-mouse AF-555-conjugated (A21424, 1:1000, Invitrogen, Waltham, MA, USA; red; NIS-only staining), or goat anti-rabbit AF-488-conjugated (A11008, 1:1000, Invitrogen, Waltham, MA, USA; green; CD44-only staining), or AF-555- and Ad hexon FITC-conjugated (AB1056F, 1:1000, Millipore-Sigma, Burlington, MA, USA; green; costaining of NIS and Ad hexon proteins) secondary antibodies. For CD44 costaining with NIS, a combination of goat anti-rabbit AF-488 secondary antibody (for CD44, A11008, 1:1000, Invitrogen, Waltham, MA, USA; green) and goat anti-mouse AF-568 secondary antibody (for NIS, A11004, 1:1000, Invitrogen, Waltham, MA, USA; red) was used. For CD44 costaining with Ad hexon, a combination of goat anti-rabbit AF-568 secondary antibody (for CD44, A11011, 1:1000, Invitrogen, Waltham, MA, USA; red) and Ad hexon FITC-conjugated (AB1056F, 1:1000, Millipore-Sigma, Burlington, MA; green) was used. Cells were washed with PBS (Corning, New York, NY, USA) again and counterstained with a nuclear stain (DAPI, Sigma-Aldrich, St. Louis, MO, USA, 0.1 µg/mL, 20 min incubation, room temperature, in the dark) just prior to image capture using a fluorescent microscope (EVOS FL Auto, Life Technologies, Carlsbad, CA, USA). Plug-in functions of ImageJ software (version 1.53t, NIH, Madison, WI, USA) were used to quantify NIS and Ad hexon expression in cells using % area measurement and normalized to DAPI area.

### 2.6. Gene Expression Analysis

Total RNA was isolated from cells using an RNeasy^®^ Mini Kit System (QIAGEN, Hilden, Germany). RNA quantity and purity were assessed with a Nanodrop Lite spectrophotometer (Thermo Fisher Scientific, Waltham, MA, USA). Total RNA (500 ng) was reverse-transcribed into cDNA using a PrimeScript™ RT Master Mix kit (TaKaRa Bio, Kusatsu, Japan). Real-time quantitative RT-PCR was performed with the SYBR Green fluorescent dye method using PowerUp™ SYBR™ Green Master Mix (Applied Biosystems, Waltham, MA, USA) and a LightCycler^®^ 480 II Real-Time PCR system (Roche, Basel, Switzerland). Primer pairs for each transcript (hNIS Forward 5′-GTAGAAGACCTCATCAAACCT-3′, hNIS Reverse 5′-GGAGCCCTGAAGGACACCTC-3′, GAPDH Forward 5′-CAACTACATGGTTTACATGTTCCAA-3′, GAPDH Reverse 5′-GCCAGTGGACTCCACGACGT-3′) were chosen with IDT SciTools (PrimerQuest™ program, IDT, Coralville, Iowa, USA. https://www.idtdna.com/SciTools, accessed on 9 September 2021) and “blasted” with NCBI (http://www.ncbi.nlm.nih.gov/BLAST/, accessed on 9 September 2021). Amplification curves were read with LightCycler^®^ 480 SW 1.5.1 software (Roche, Basel, Switzerland) using the comparative cycle threshold method. The steady-state level of mRNA for each gene of interest was normalized against the value for GAPDH mRNA.

### 2.7. Tumorsphere Formation Assay

Single-cell suspensions of parental MCF-7 and paclitaxel-resistant TaxR cells were filtered through a 40 µm sieve (BD Falcon, Franklin Lakes, NJ, USA) and seeded in 24-wells ULA plates (#3473, Neta Scientific, Hainesport, NJ, USA) at 1000 cells per well. Cells were grown in MEBM medium (#CC-3151, Lonza, Basel, Switzerland) without serum, supplemented with 4 mg/mL of insulin (Thermo Fisher Scientific, Waltham, MA, USA), 10 ug/mL hydrocortisone (Sigma-Aldrich, St. Louis, MO, USA), 1 mL B27 supplement (Thermo Fisher Scientific, Waltham, MA, USA), 100 µg/mL EGF (Sigma-Aldrich, St. Louis, MO, USA), and 0.35 µL of beta-mercaptoethanol (Sigma-Aldrich, St. Louis, MO, USA). The medium was then mixed with 500 mg methylcellulose (Thermo Fisher Scientific, Waltham, MA, USA) diluted in MEBM supplemented with the previously mentioned derivatives at a 1:1 ratio. Tumorspheres were allowed to form in suspension for 7 days. Cells were infected with OAd either on the seeding day or at the end of the 7 days’ formation. Tumorsphere images were taken using an All-In-One microscope BZ-X800 (Keyence Corporation, Osaka, Japan) using brightfield at 4× magnification. Edge points of the wells were selected to capture the tumorspheres in each well. Images were stitched using BZ-X800 Analyzer software (Keyence Corporation, Osaka, Japan). Tumorspheres were analyzed by total number and scored by manual counting using a scaled grid on ImageJ software (version 1.53t, NIH, Madison, WI, USA). Data are presented as the average ± SD of four independent measurements.

### 2.8. Statistical Analysis

Statistical analyses were conducted with GraphPad Prism 6 software (LaJolla, CA, USA). Data were expressed as mean ± standard deviation (SD). Student’s unpaired two-tailed *t*-test was used to determine statistical significance between groups when the distribution of the samples in a given condition was normal. Normal distribution of samples was evaluated by two normality tests (D’Agostino and Pearson omnibus and Shapiro–Wilk). For groups that did not pass the normality test, nonparametric statistical comparisons were conducted using the Mann–Whitney U test or the one-way ANOVA with post hoc analysis if more than two groups were compared. A value of *p* < 0.05 was considered statistically significant (* *p* < 0.05; ** *p* < 0.01, *** *p* < 0.001, **** *p* < 0.0001 versus control).

## 3. Results

1. Superiority of genetically modified Ad5/3 fiber for human breast cancer cells.

**Figure 1:**
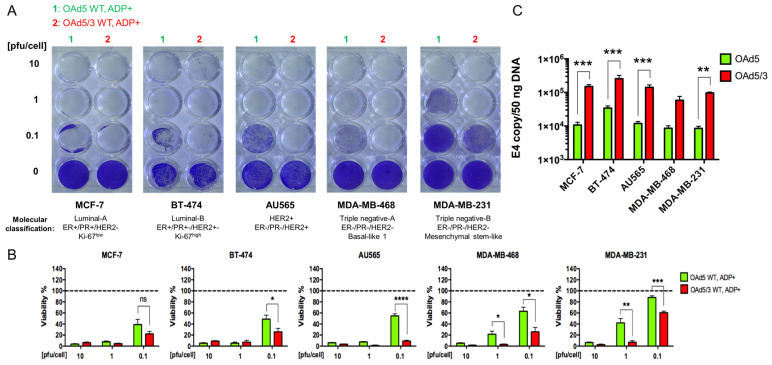
Ad5/3-modified fiber improved OAd replication and binding with breast cancer cells. (**A**) Crystal violet staining of human breast cancer cell lines from heterogenous molecular classification and (**B**) associated density quantification analysis of the percentage of remaining living cells compared to untreated control, 5 days postinfection with OAd displaying either Ad5- or Ad5/Ad3-modified fiber, showed superior replication with Ad5/3-modified OAd vector. (**C**) Binding assay demonstrated that amount of Ad E4 copies, represented on a log scale with base 10, was far superior in all tested cell lines with a virus harboring the Ad5/3-modified fiber (OAd5/3) compared to the fiber-unmodified control (OAd5). Data are expressed as the amount of E4 copy numbers in a normalized amount of total DNA extracted from breast cancer cell lines. The statistical significance was determined by a one-way ANOVA with post hoc analysis. Data are expressed as mean with error bars representing standard deviation calculated from three replicates: * *p* < 0.05, ** *p* < 0.01, *** *p* < 0.001, **** *p* < 0.0001. WT: wild type replication controlled.

In order to determine if the Ad5/Ad3-modified fiber is effective in redirecting the OAd from CAR and improving its attachment to and cytolytic potential in breast cancer, we took a heterogeneous population of human breast cancer cell lines from different molecular classifications, covering the complete spectrum of human disease. Crystal violet assay evaluation showed that 5 days after infection, the replication ability of the Ad5/3-modified OAd vector (OAd5/3 WT, ADP(+)) was superior by at least one order of pfu per cell unit to the Ad5-unmodified fiber (OAd5 WT, ADP(+)) counterpart in all tested cell lines (Figure 1A). While not significative in the MCF-7 cell line, viability quantification confirmed a trend of superior killing effect with the Ad5/3-modified OAd vector compared to the Ad5-unmodified-fiber counterpart (Figure 1B). In addition, after incubating cells for 2 h with virus at 4 °C, which allows virus binding but prevents internalization into the cells, we determined that the binding ability of the Ad5/3 fiber-modified vector (OAd5/3) was significantly improved compared to that with the identical fiber-unmodified control (OAd5) in all tested breast cancer cell lines (Figure 1C). These two complementary findings indicate that chimeric Ad5/3 fiber significantly outperformed Ad5 binding and oncolytic abilities in all tested human breast cancer cell lines, regardless of their molecular classification.

2. The effect of Cox-2 promoter and ADP deletion on OAd-hNIS replication and killing ability.

**Figure 2:**
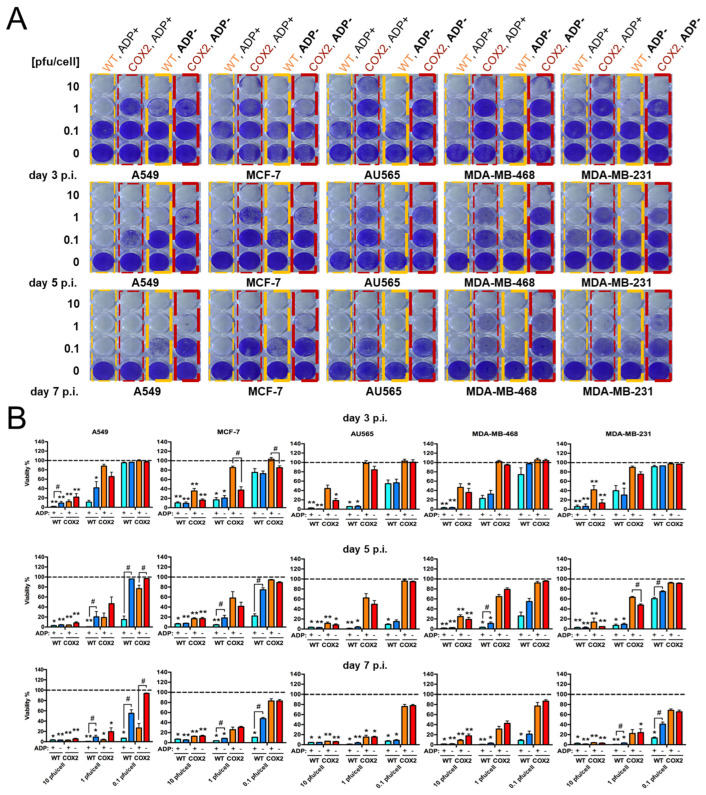
OAd5/3-hNIS vectors can replicate and lyse breast cancer cells upon low titer infection. ADP deletion does not significantly impact oncolysis. (**A**) Crystal violet staining of human breast cancer cell lines and (**B**) associated density quantification analysis of the percentage of remaining living cells compared to untreated control. Oncolytic ability of OAd5/3-hNIS vectors was evaluated over time at 3, 5, and 7 days postinfection with 3 viral titers of 10, 1, and 0.1 pfu/cell. The A549 cell line was used as standard CAR+, Ad3-receptor+, and Cox-2+ control. The statistical significance was determined by a one-way ANOVA with post hoc analysis. Data are expressed as mean with error bars representing standard deviation calculated from four replicates. Compared to the untreated control group, * *p* < 0.05, ** *p* < 0.01. Compared to the ADP-deleted counterpart, # *p* < 0.05.

After determining the superiority of the OAd with Ad5/3 fiber, we compared Cox-2-controlled OAd-NIS vectors versus their identical nonselective counterparts (wild-type (WT) replication vectors). We also evaluated the effect of ADP deletion on the OAd oncolytic potential. The cancer cells were infected at intermediate (10), low (1), and very low (0.1) titers of pfu per cell to allow at least a few rounds of virus replication for the best evaluation of the viral progeny production. Surviving cells were analyzed by crystal violet staining (Figure 2A) and corresponding density quantification (Figure 2B). Uniformly, vectors with Cox-2-regulated replication demonstrated effective oncolysis effect, which was slightly less efficient than their WT replication vector counterparts (see statistical analysis in Table S1). Similar to breast cancer cells, Cox-2-regulated vectors were less efficient than WT replication counterparts at killing A549 cells (used here as a Cox-2-positive control).

Regarding ADP deletion, ADP(−) viruses could kill all tested breast cancer cell lines in a similar manner to their ADP(+) counterparts when vectors were Cox-2-regulated. On the other hand, ADP(+) WT replication vectors could kill more efficiently than their ADP(−) counterparts in all tested breast cancer cell lines, with the only exception being AU565 cells. The replication efficacy of ADP(−) vectors was especially visible when comparing Cox-2-regulated vectors with WT replication vectors in MCF-7, MDA-MB-468, and MDA-MB-231 cell lines (Figure 2A,B). Interestingly, while ADP deletion in Cox-2-regulated vectors showed no significative effect on the killing ability in all tested breast cancer cell lines (Figure 2B), it was able to slow adenovirus replication in A549 control lung adenocarcinoma cells, which could be attributed to different “compensation” mechanisms intrinsic to breast cancer cells. Overall, these data indicate that OAd5/3 Cox-2 hNIS vectors can efficiently replicate and lyse breast cancer cells in vitro at low titer and that ADP deletion is not a significant factor in reducing the oncolytic potential in breast cancer cells.

3. The effect of ADP deletion on hNIS expression in breast cancer cells.

**Figure 3:**
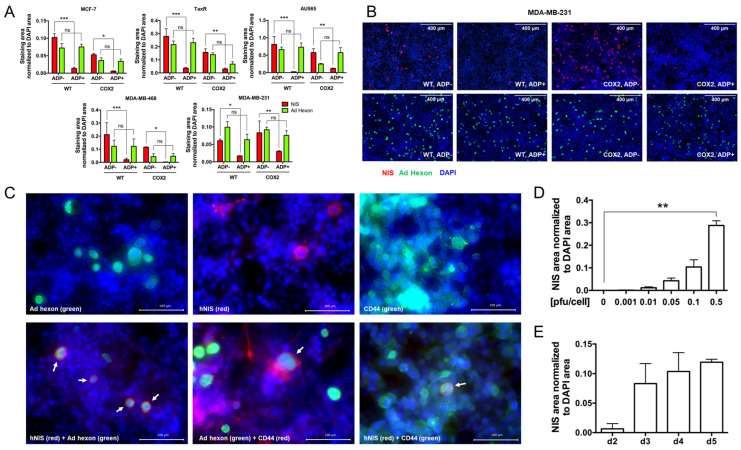
ADP deletion improves hNIS expression in breast cancer cells in a time- and dose-dependent manner without significantly impairing virus replication. (**A**) Quantification of staining area (hNIS in red, Ad hexon protein in green) normalized to DAPI area in MCF-7, TaxR, MDA-MB-231, MDA-MB-468, and AU565 human breast cancer cells after 4 days postinfection with OAd5/3-hNIS vectors at 0.1 pfu/cell showed improved hNIS transgene expression in ADP-deleted vectors (OAd5/3 WT ADP(−) hNIS and OAd5/3 Cox2 ADP(−) hNIS) compared to ADP-conserved vectors (OAd5/3 WT ADP(+) hNIS and OAd5/3 Cox2 ADP(+) hNIS). (**B**) Representative immunofluorescence images of hNIS (red) and Ad hexon protein (green) expression, counterstained with DAPI (blue) in MDA-MB-231 cells infected for 4 days with 0.1 pfu/cell of OAd5/3-hNIS vectors. (**C**) Magnification at 40× of merge single or costaining of hNIS (red), Ad hexon protein (green), cell-surface marker CD44 (red if costained with Ad hexon protein or green if costained with hNIS or alone), and DAPI (blue) in MDA-MB-231 cells infected for 4 days with 0.1 pfu/cell of OAd5/3 Cox-2 ADP(−) hNIS. (**D**) Dose-dependent quantification of hNIS area normalized to DAPI area in MDA-MB-231 cells infected with OAd5/3 Cox-2 ADP(−) hNIS for 4 days. (**E**) Time-dependent quantification of hNIS area normalized to DAPI area in MDA-MB-231 cells infected with OAd5/3 Cox-2 ADP(−) hNIS at 0.1 pfu/cell. The statistical significance was determined by a one-way ANOVA with post hoc analysis. Data are expressed as mean with error bars representing standard deviation calculated from three replicates: * *p* < 0.05, ** *p* < 0.01, *** *p* < 0.001.

To understand the impact of ADP on hNIS expression, we performed immunofluorescence quantification analyses of MCF-7 (luminal-A), TaxR (luminal-A, paclitaxel-resistant), AU565 (HER2+), MDA-MB-468 (triple negative-A), and MDA-MB-231 (triple negative-B) cells infected with OAd5/3-hNIS ADP(+) and ADP(−) vectors (Figure 3A). In representative images from MDA-MB-231, Ad hexon protein expression was observed after infection with all hNIS-expressing viruses; however, hNIS expression was higher in cells infected with ADP(−) vectors (Figure 3B). Indeed, after quantification analysis, while Ad hexon protein expression remained unaffected by ADP deletion, it significantly improved levels of NIS in the five tested breast cancer cell lines (Figure 3A). Higher magnification of OAd5/3 Cox-2 ADP(−) hNIS-infected MDA-MB-231 cells, costained for hNIS and Ad hexon proteins, showed internal localization of Ad hexon protein, while hNIS was more localized to the cytoplasmic membrane, where it could be functional (Figure 3C). Furthermore, costaining with the cell-surface protein marker CD44 showed how hNIS was localized in the cytoplasmic membrane, while Ad hexon protein transiently localized to both the nucleus and the cytoplasm (Figure 3C). While it was not significant, we found that hNIS expression gradually increased over time in MDA-MB-231 cells (Figure 3E) and significantly in a dose-dependent manner (Figure 3D) upon infection with OAd5/3 Cox-2 ADP(−) hNIS. Overall, these analyses revealed that the deletion of ADP greatly improved the expression of hNIS in breast cancer cells, most likely due to greater hNIS membrane localization, as a result of lesser ADP activity that disrupted the cytoplasmic membrane during the adenoviral lytic phase.

4. Evaluation of OAd5/3-hNIS vectors’ selectivity.

**Figure 4:**
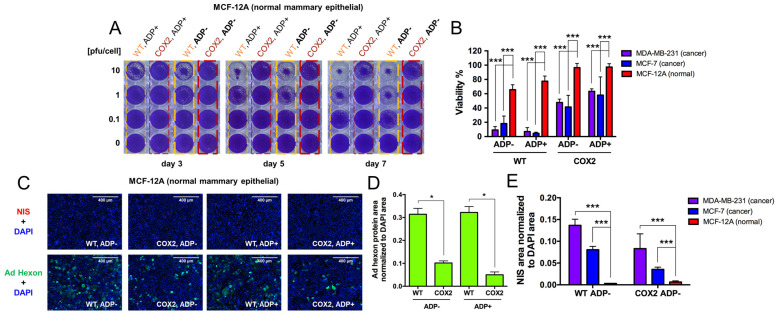
Preferential replication of Cox-2 promoter-controlled vectors. (**A**) Cox-2-controlled vectors show minimum replication compared to WT replication vectors in normal human epithelial breast cells MCF-12A. Replication potential was shown by crystal violet staining at 3, 5, and 7 days postinfection with 3 viral titers of 10, 1, and 0.1 pfu/cell. (**B**) The percentage of viability compared to control (untreated) wells was determined from crystal violet staining density quantification after 5 days postinfection with 1 pfu/cell in MCF-12A breast cells and MCF-7 and MDA-MB-231 breast cancer cells. (**C**) Representative immunofluorescence images of hNIS (red) and Ad hexon protein (green) expression, counterstained with DAPI (blue) in MCF-12A cells infected for 3 days with 0.1 pfu/cell of OAd5/3-hNIS vectors. (**D**) Ad hexon protein area quantification normalized to DAPI area from immunofluorescence staining of MCF-12A, 3 days postinfection with 0.1 pfu/cell. (**E**) Comparison of hNIS expression from immunofluorescence area quantification in MCF-12A, MCF-7, and MDA-MB-231 cells, 3 days postinfection with OAd5/3 WT or Cox-2 ADP(−) hNIS at 0.1 pfu/cell. The statistical significance was determined by a one-way ANOVA with post hoc analysis. Data are expressed as mean with error bars representing standard deviation calculated from four replicates: * *p* < 0.05, *** *p* < 0.001.

In an effort to determine the specificity and, by association, safety of our hNIS-expressing replication-competent vectors, we evaluated the killing and replication abilities of OAd5/3-hNIS viruses in normal human epithelial breast cells MCF-12A compared to those of human breast cancer cell lines. After crystal violet assay, we observed very limited killing potential of the viruses with WT-regulated replication and, furthermore, no killing ability of Cox-2-regulated replication viruses, even at a later time point with a titer of 10 pfu/cell (Figure 4A). When analyzing the remaining viability, all vectors displayed significant killing effect after 5 days postinfection in MDA-MB-231 and MCF-7 breast cancer cell lines compared to normal breast cell line MCF-12A (Figure 4B). Accordingly, with the crystal violet staining, we found the evidence of some viral replication in MCF-12A cells since they expressed Ad hexon proteins after immunofluorescence staining (Figure 4C), which was significantly improved with WT-regulated replication vectors compared to their Cox-2-regulated replication counterparts (Figure 4D). However, hNIS expression was not detectable in MCF-12A cells (Figure 4C), indicating a noneffective replication of the virus in normal breast cells. This is further supported by quantification analysis of hNIS area expression in MCF-12A compared to MDA-MB-231 and MCF-7 breast cancer cells upon infection with ADP-deleted vectors (bearing either WT- or Cox-2-regulated replication) (Figure 4E).

5. Evaluation of OAd-hNIS vectors’ replication potential in chemoresistant TaxR cells.

**Figure 5:**
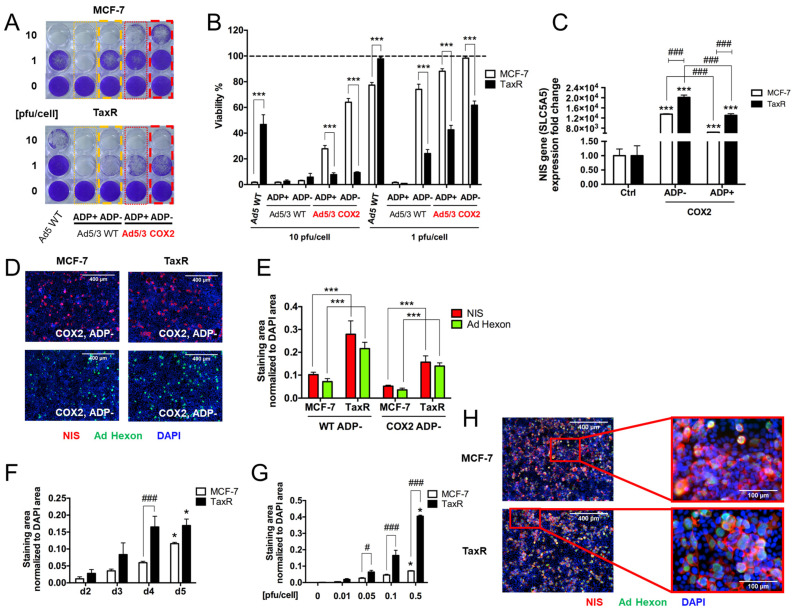
Paclitaxel-resistant ER+ MCF-7 cells are more responsive to OAd-hNIS infection than parental MCF-7 cells. (**A**) Crystal violet staining and (**B**) associated density quantification analysis of the percentage of remaining living cells compared to untreated control of human breast cancer cells MCF-7 (parental, chemosensitive) and the paclitaxel-resistant MCF-7 subclone (TaxR) on day 4 postinfection with OAd5/3-NIS vectors. (**C**) qPCR analysis of SLC5A5 gene expression, normalized to GAPDH, in TaxR and MCF-7 cells after 48 h postinfection with 0.5 pfu/cell of OAd5/3 Cox-2 hNIS vectors. (**D**) Representative immunofluorescence imaging of hNIS (red) and Ad hexon protein (green) areas, counterstained with DAPI (blue) in MCF-7 and TaxR cells, 4 days postinfection with 0.1 pfu/cell of OAd5/3 Cox-2 ADP(−) hNIS. (**E**) Quantification of staining area (NhIS in red, Ad hexon protein in green) normalized to DAPI area in MCF-7 and TaxR cells after 4 days postinfection with OAd5/3 WT ADP(−) hNIS or OAd5/3 Cox-2 ADP(−) hNIS at 0.1 pfu/cell. (**F**) Comparison of time-dependent quantification of hNIS area normalized to DAPI area in MCF-7 and TaxR cells infected with OAd5/3 Cox-2 ADP(−) hNIS at 0.1 pfu/cell. (**G**) Comparison of dose-dependent quantification of hNIS area normalized to DAPI area in MCF-7 and TaxR cells infected with OAd5/3 Cox-2 ADP(−) hNIS for 4 days. (**H**) Higher magnification (10X left, 40X right) of merge staining of hNIS (red), Ad hexon protein (green), and DAPI (blue) in MCF-7 and TaxR cells infected for 5 days with 0.5 pfu/cell of OAd5/3 Cox-2 ADP(−) hNIS. The statistical significance was determined by a one-way ANOVA with post hoc analysis. Data are expressed as mean with error bars representing standard deviation calculated from three replicates. Compared to the corresponding control group (untreated), * *p* < 0.05, *** *p* < 0.001. Compared either to ADP-deleted vector or compared to the same condition between MCF-7 and TaxR cells, # *p* < 0.05, ### *p* < 0.001. Ctrl: untreated control. TaxR cells are always cultured in the presence of 2 mM paclitaxel.

Since we observed effective binding, replication, and hNIS expression in hormone receptor-positive breast cancer cell lines upon OAd5/3 ADP(−) hNIS infection, we wanted to know if the virus could still be effective in chemoresistant ER+ breast cancer cells. Dr. Ostrander’s laboratory supplied us with paclitaxel-resistant (TaxR) and paclitaxel-sensitive parental MCF-7 cells. The TaxR cells were previously characterized as exhibiting features and markers of breast cancer stem cells, a subpopulation of cells responsible for ER+ breast cancer late metastatic recurrences [27]. Since breast cancer stem cells are usually slowly proliferative cells, it was highly probable that our OAd-hNIS vectors would less efficiently replicate in those cells. Surprisingly, we found that while Ad5 WT virus replicated better in MCF-7 than TaxR cells, all OAd5/3 vectors more efficiently killed TaxR cells than MCF-7 cells after crystal violet analysis, especially when using vectors under the Cox-2 promoter (Figure 5A). These findings were confirmed to be significant upon viability analysis of crystal violet staining (Figure 5B). In accordance with these initial findings, we found that hNIS gene expression was significantly improved in TaxR cells compared to MCF-7 cells for the same viral vector (Figure 5C). In addition to the gene expression increase, both the levels of hNIS and Ad hexon proteins were significantly improved in TaxR compared to MCF-7 cells (Figure 5D,E) after immunofluorescence analysis. Interestingly, when we compared the expression of hNIS area in MCF-7 cells and TaxR cells under the same infection condition, we found that compared to MCF-7 cells, it was generally improved in TaxR cells at four different time points, with a significantly increased expression on day 4 postinfection (Figure 5F), and with three different titers of 0.05, 0.1, and 0.5 pfu/cell (Figure 5G) tested on the same day postinfection with OAd5/3 Cox-2 ADP(−) hNIS. Higher magnification of MCF-7 cells and TaxR cells costained with Ad hexon and hNIS proteins showed a less diffuse, improved cellular membrane localization of hNIS in TaxR cells compared to MCF-7 cells after infection with OAd5/3 Cox-2 ADP(−) hNIS (Figure 5H). These results show the potential of OAd to efficiently treat chemoresistant breast cancer.

6. Evaluation of OAd5/3 Cox-2 ADP(−) hNIS efficiency to target paclitaxel-resistant BCSCs in tumorsphere assay.

**Figure 6:**
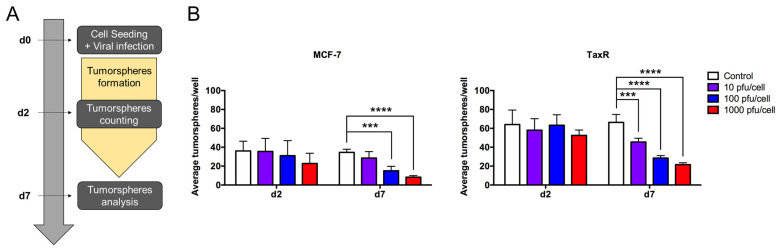
OAd5/3 Cox-2 ADP(−) hNIS effectively inhibits tumorsphere formation in MCF-7 and TaxR breast cancer stem cell models. (**A**) Workflow of the experiment. (**B**) Tumorspheres assay in MCF-7 and TaxR cells with infection upon generation with OAd5/3 Cox-2 ADP(−) hNIS shows change in average number of tumorspheres over time and increasing viral titers. The statistical significance was determined by a one-way ANOVA with post hoc analysis. Data are expressed as mean with error bars representing standard deviation calculated from four replicates: *** *p* < 0.001, **** *p* < 0.0001.

**Figure 7:**
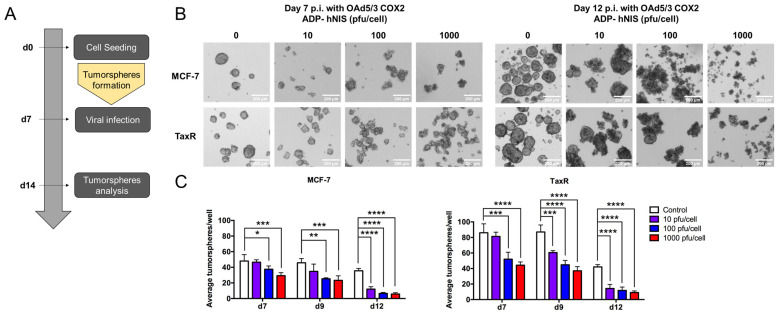
OAd5/3 Cox-2 ADP(−) hNIS effectively kills tumorsphere from breast cancer stem cell in MCF-7 and TaxR model. (**A**) Workflow of the experiment. (**B**) Tumorspheres assay in MCF-7 and TaxR cells exhibits modified spheres morphology upon OAd5/3 Cox-2 ADP(−) hNIS infection over time and increasing viral titers and (**C**) changes in the average number of tumorspheres upon viral infection (d7–d12). The statistical significance was determined by a one-way ANOVA with post hoc analysis. Data are expressed as mean with error bars representing standard deviation calculated from four replicates: * *p* < 0.05, ** *p* < 0.01, *** *p* < 0.001, **** *p* < 0.0001.

While it is surprising that the killing and replicative properties of OAd5/3-hNIS vectors were improved in vitro in chemoresistant breast cancer stem cells compared to parental cells, it is possible that 2D monolayer cultures allowed for the faster division of TaxR cells, resulting in more efficient Ad replication. To confirm these findings, we analyzed the efficacy of our vectors in 3D cultures by generating tumorspheres from MCF-7 (chemosensitive) and TaxR (chemoresistant) subcultures. These cells were grown in ultra-low-attachment plates in the absence of serum, which allowed the breast cancer stem cells to divide and form spheres. We started by infecting the cells with OAd5/3 Cox-2 ADP(−) hNIS virus at different titers alongside the tumorsphere initiation (Figure 6A). On day 7 postinfection with a titer as low as 10 pfu/cell, we observed a significant decrease in the number of tumorspheres generated by TaxR cells compared to the untreated control (Figure 6B). After determining that OAd5/3 Cox-2 ADP(−) hNIS can inhibit tumorsphere formation, we investigated its strength to lyse preformed tumorspheres enriched with BCSCs by employing a more clinically relevant setting. In this assay, we infected already formed tumorspheres seven days after their initiation. We observed changes in the tumorspheres’ morphology at days 7 and 12 postinfection as the titer increased (Figure 7A). On day 7, apoptotic bodies were observed around tumorspheres in 10 and 100 pfu/cell conditions, while infection with the higher titer (1000 pfu/cell) resulted in apoptotic cell debris formation with smaller spheres. On day 12, the spheres were bigger in the untreated controls, but upon infection with 10 and 100 pfu/cell, the apoptotic bodies merged to form larger cellular clumps with a nondefined morphology. Similar to day 7, infection with 1000 pfu/cell resulted in complete tumorsphere destruction with only cell debris remaining. As expected, in both experiments (Figure 6B and Figure 7B), the untreated TaxR cells generated more tumorspheres than the parental MCF-7. This could be explained by the fact that the TaxR cells contain more CD44^hi^/CD24^lo^ cells than the parental MCF-7. However, the number of tumorspheres dropped significantly more in TaxR cells than MCF-7 cells, especially on days 7 and 12 postinfection. We concluded that the OAd5/3-hNIS vector can both inhibit tumorsphere formation and kill preformed tumorspheres from MCF-7 and TaxR models, with a stronger effect visible in the BCSC-enriched TaxR chemoresistant cells.

7. Evaluation of OAd5/3 Cox-2 ADP(−) hNIS in combination treatment with paclitaxel on chemoresistant and chemosensitive ER+ BCSCs.

**Figure 8:**
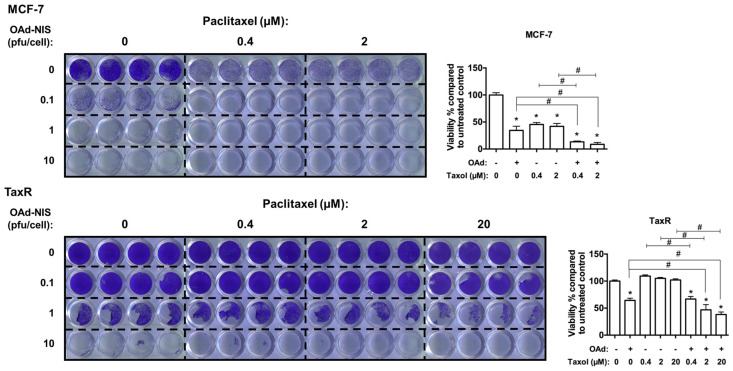
OAd-hNIS reconditioned the status of chemoresistant ER+ breast cancer stem cells into paclitaxel-sensitive cells. Crystal violet assay and associated density quantification in MCF-7 (0.1 pfu/cell of OAd for reported quantification) and TaxR (1 pfu/cell of OAd for reported quantification) cells cotreated with increasing single dose of paclitaxel and OAd5/3 Cox-2 ADP(−) hNIS for 5 days. The statistical significance was determined by a one-way ANOVA with post hoc analysis. Data are expressed as mean with error bars representing standard deviation calculated from four replicates. Compared to the untreated control group, * *p* < 0.05. Compared between single and combination groups, # *p* < 0.05.

We wanted to determine why our OAd-hNIS vectors were more efficient in chemoresistant cells than parental chemosensitive cells. To investigate this, we performed a cotreatment of both MCF-7 cells and TaxR cells with OAd5/3 Cox-2 ADP(−) hNIS and an increasing dose of paclitaxel (Figure 8). In MCF-7 cells, the presence of paclitaxel alone killed more than 50% of cells, and the virus alone at 0.1 pfu/cell could kill up to 60% of the cells. When cotreated together, the viability significantly dropped to 10%, confirming an additive effect in these chemosensitive cells. In TaxR cells, not surprisingly, the paclitaxel alone could not kill the cells, even at a high dose of 20 µM, and the virus at 1 pfu/cell killed 40% of cells. Surprisingly, when TaxR cells were cotreated with virus and paclitaxel, we found that OAd-hNIS sensitized TaxR cells to paclitaxel upon virus infection, achieving up to a 60% killing effect. These data demonstrate that by an unknown mechanism, OAd can modify the behavior of chemoresistant breast cancer stem cells, promising huge potential for the treatment of ER+ metastatic recurrences.

## 4. Discussion

The overall scope of our study was to optimize and validate the structure of NIS-based vectors tailored specifically to breast cancer cells through rigorous in vitro investigations. This preclinical assessment of the efficacy of our various hNIS-expressing OAd vectors will aid in identifying the most promising modifications, potentially paving the way for a potent therapeutic strategy against therapy-resistant subsets of advanced breast cancers. Key experimental highlights include the following: (1) The enhancement of OAd’s infectivity by targeting overexpressed breast cancer receptors and utilization of a tissue-specific promoter. (2) Evaluation of the transgene hNIS expression, which allows for the selective uptake of radioactive iodine by cancer cells. (3) The employment of a cutting-edge model of ER+ paclitaxel-resistant breast cancer originating from the MCF-7 cell line (TaxR) to accurately mimic the challenges of therapy-resistant breast cancer. These cells not only display resistance to paclitaxel but also are enriched with BCSC markers, making them an ideal platform for our investigations. (4) To comprehensively assess OAd’s potential to target BCSCs, we employed 3D tumorsphere cultures. These cultures provided a dynamic environment that closely resembled the physiological conditions encountered in breast cancer, allowing us to evaluate OAd’s ability to disrupt BCSC populations effectively. Our research is rooted in the urgent need to address the persistent challenges posed by hormone receptor-positive breast cancer, which, despite substantial progress in treatment modalities, remains a formidable adversary, especially in its metastatic and therapy-resistant forms.

Despite advances in breast cancer diagnosis and treatment, many patients still experience relapse, resulting in disease progression, recurrence, and reduced overall survival. Much focus has been put on the intrinsic subtyping based in the presence (or absence) of markers such as ER, PR, and HER2. Additionally, it is widely understood that tumors are also composed of heterogeneous populations of cells containing immune cells, fibroblasts, and cancer stem cells. In breast tumors, the cells in the small population displaying stemlike properties are known as BCSCs. While several potential BCSC markers have been identified, this rare population tends to exhibit a CD44^hi^/CD24^lo^ phenotype with high ALDH activity (ALDH+) and has been shown to form colonies in tumorsphere assays [27], part of the self-renewal feature of stem cells. Because of their higher tolerability to chemotherapy, hormone therapy, and radiotherapy [28], these cells can reproduce the bulk of the tumor after a reduction in cell populations sensitive to first-line therapy, leading to disease relapse. Significant advances have been made in the identification, isolation, and characterization of BCSCs, and as a consequence, the development of new compounds targeting this small cell population are being developed [29]. However, the selection pressure of monotherapy could generate resistant BCSC clones, which is why we believe a combination therapy is more desirable. In that regard, since the plasticity of BCSCs allows them to shift between stemlike and non-stemlike states, the targeted therapy must not be restricted to this small population but rather also must address more differentiated progenitors and the bulk tumor cell population.

OAds are a promising therapy tool for these challenging cancer cells. Their ability to selectively target, replicate, and lyse most cancer cells, as well as to restore antitumor immunity in cancer patients, has been well demonstrated in many reports and clinical studies [30,31]. They can also be “armed” with anticancer transgenes to enhance efficacy with a multimodal mechanism of action [18,32,33]. Several preclinical studies have shown promising results in using OAds to treat breast cancer, with the potential to improve survival rates and reduce side effects compared to traditional treatments [34].

As of today, there are more than a dozen OAd-based vectors that were or are currently under investigation for breast cancer treatments including Ad3-hTERT-E1A [35], Onyx-015 [36], Telomelysin [37], ColoAd1 [38], VCN-01 [39], H103 [40], VISTA [41], Ad HSV-tk [42], Ad5/3 delta 24 GMCSF [43,44], Ad5/3 E2F delta 24 GMCSF [45], Ad5 delta 24 GMCSF [46,47], Ad5 RGD delta 24 GMCSF [48], and ICOVIR-7 [49]. However, while demonstrating certain efficacity in breast cancer models, those vectors were not strategically designed to specifically target and eradicate therapy-resistant BCSCs. These clinical trials have also underlined the challenges for successful oncolytic virotherapy, which include promoting efficient intratumoral spread (by overcoming poor viral replication) and assuring production of high levels of functional therapeutic transgenes, which could then be employed for combination therapy. Our vector (OAd5/3 Cox-2 ADP(−) hNIS) was specifically refined to address these issues.

For oncolytic virotherapy, and Ad in particular, poor tumor transduction remains an obstacle, as many cancer cells are deficient for the primary Ad receptor, CAR. The generation of infectivity-improved Ad, which can bind to cells via CAR-independent mechanisms, can greatly improve adenoviral entry and infectivity. For example, a LyP-1 (p32) peptide was inserted into the Ad fiber to improve binding and infectivity in breast cancer tissues [50]. The other common approach to redirect OAds from CAR is based on the genetic modification of Ad fiber to switch the Ad5 fiber knob to the knob derived from a different serotype. The improved infectivity of Ad5/3 chimeric fiber, which recognizes Ad3 receptors (CD46 and DSG2), was demonstrated in different cancer models, including breast cancer [51], glioma [52], esophageal adenocarcinoma [53], and pancreatic cancer [32,54].

In this study, we employed a diverse collection of human breast cancer cell lines encompassing various molecular classifications. This allowed us to investigate the effectiveness of the Ad5/Ad3-modified fiber in retargeting and enhancing OAd attachment to breast cancer cells, covering the entire spectrum of human breast cancer disease. Among these cell lines, MCF-7 (luminal-A) and BT-474 (luminal-B with HER2 expression) are luminal-class breast cancer cells characterized by the expression of hormonal receptors (estrogen and progesterone). The AU565 cell line belongs to the HER2+ classification. On the other hand, the MDA-MB-468 and MDA-MB-231 cell lines are classified as triple-negative, lacking expression of both hormonal receptors and HER2. We demonstrated that the Ad5/3-modified fiber significantly improved Ad binding to all tested human breast cancer cell lines compared to the control Ad5 fiber. What was even more interesting to observe was that the Ad5/3-fiber-modified OAd-hNIS vectors demonstrated greater replication in paclitaxel-resistant cells compared to the parental cells. Of note, the control vector with Ad5 fiber (OAd5 WT) was more efficient in killing MCF-7 parental cells than the paclitaxel-resistant BCSCs TaxR. We hypothesized that it could be due to the lesser expression of CAR in stem cells compared to the parental tumor cells. These findings correlate well with previous reports demonstrating the overexpression of CD46 and DSG2 in human breast cancer tissue, especially in patients with unfavorable prognoses [22,23]. Overall, our data further support the application of Ad5/3 genetically modified vectors for the treatment of challenging breast cancer cases.

Past clinical trials have revealed the limited efficacy of oncolytic viruses when used in monotherapy [35,36,37,38,39,40,41,42,43,44,45,46,47,48,49]. To enhance their antitumor effects, a powerful strategy involves combining them with other therapies. In our approach, we equipped the Ad with the hNIS transgene, a molecule with immense potential for molecular imaging and targeted radionuclide therapy. NIS has a decades-long successful history in thyroid cancer treatment, where it mediates cellular iodine uptake [16,55]. The application of oncolytic viruses carrying the NIS gene for nonthyroid cancers has garnered significant interest in recent years, owing to their ability to deliver NIS specifically to tumor sites [18,51,56,57,58,59]. Further, this approach holds great promise as it can be readily translated into clinical settings. Indeed, SPECT/CT and PET/CT scanners are already being routinely used as a staging tool in advanced cancers for breast cancer patients, offering highly accurate localization and quantitative assessment of radioactivity [18,56,57,60].

Our laboratory has previously demonstrated the potential of OAd vectors expressing hNIS for imaging and therapy in cancers beyond breast cancer [17,18]. Some earlier studies explored Ad expressing NIS for combination radiotherapy in breast cancer [51,61,62,63]; however, they underscored the necessity of achieving sufficient levels of the NIS transgene delivery to cancer cells to fully realize the imaging and radiotherapy potential.

In order to improve hNIS expression, we encoded the hNIS gene into the Ad E3 region while deleting the ADP from the same region. The expression of a transgene from E3 is controlled by the adenovirus major late promoter and is consistent with the replication cycle—a property that allows for continuous expression of hNIS at each round of viral replication [64]. Studies have also shown that deleting nonessential E3 region genes while maintaining ADP resulted in higher levels of ADP expression, improving oncolytic potential [18,51,65]. Since then, ADP overexpressing vectors have been used by many with the goal to improve the oncolytic potential of Ad-based therapeutics. However, previous research conducted in our laboratory demonstrated that ADP expression negatively affects hNIS expression [18]. Consistent with this finding, our data in breast cancer cells showed that ADP expression negatively affects hNIS expression. Thus, in five tested breast cancer cell lines (MCF-7, TaxR, AU565, MDA-MB-468, and MDA-MB-231), the protein levels of NhIS assessed by immunofluorescence staining analysis were far superior in cells infected with OAd5/3-hNIS ADP(−) than ADP(+) vectors. As expected, the expression of hNIS in breast cancer cells was increased with each Ad replication cycle as we observed increased levels of hNIS in a time- and dose-dependent manner upon viral infection. We hypothesized that the deletion of ADP improves the membrane localization of hNIS by attenuating oncolysis, which is based on our immunofluorescence staining observations where hNIS expression was enhanced to the cytoplasmic membrane of MDA-MB-231 cells upon OAd5/3-hNIS ADP(−) infection.

Notably, ADP deletion did not significantly affect the oncolytic activity of the virus, as it was demonstrated by the crystal violet and Ad hexon staining in all tested breast cancer cell lines. The levels of Ad hexon protein were unchanged in breast cancer cells upon infection by either ADP(−) or ADP(+) vectors. The ADP-deleted vectors demonstrated impressive oncolysis upon extremely low titers of infection at 1 and 0.1 pfu/cell in all tested breast cancer subtypes, including HER2+, triple-negative, and estrogen receptor-positive (ER+) cells. This data corelated well with our previous study when we analyzed the effect of ADP deletion on pancreatic cancer cells [18]. Interestingly, the uncompromised oncolytic activity of ADP(−) vectors compared to ADP(+) counterparts in breast cancer cells was even more noticeable when we compared it to A549 cells, a control lung adenocarcinoma cell line. Contrary to breast cancer cells, in A549 cells, ADP deletion did significantly decrease the oncolytic potential of our virus. This could be explained by “compensation” mechanisms such as a stronger activity of the Cox-2 promoter in A549 cells and/or a better performance of the 5/3-modified fiber in breast cancer cells compared to relatively CAR-positive A549 cells.

Another major aspect for successful oncolytic virotherapy is selectivity. It is well-known that human Ad possesses an intrinsic selectivity and tends to replicate more efficiently in cancer cells than in normal tissues [66,67]. This corresponds with our observations where we have seen a greater replication and killing effect in breast cancer cells than in normal mammary cells with our WT replication OAds (OAd5/3 WT hNIS, OAd5 WT hNIS, OAd5 WT), which served as the nonselective control in this situation. However, to improve selectivity even further, one of the most used methods is the incorporation of tissue-specific promoters (TSPs) to control the Ad E1 gene expression and subsequent viral replication.

Previous reports highlighted the use of different promoters for breast cancer virotherapy such as hTERT [35,37,62,68,69,70,71], MDR [72], mucin-1 (MUC1) [51,73], hypoxia-responsive [71,74,75], estrogen-responsive [61,71,75], surviving [76], L-plastin [77], and Cyclooxygenase-2 (Cox-2) [72]. It is important to note that breast cancer is a very heterogenous disease and the choice of the TSP greatly relies on the model chosen. For example, while MUC1 is overall highly expressed in most breast cancer models, some cell lines like the triple-negative MDA-MB-231 are deficient [51].

The expression of Cox-2 in breast cancer varies in individuals based on different factors but is overall largely overexpressed. For example, in a study conducted on a cohort of 123 breast cancer patients, Cox-2 overexpression was detected in more than 90% of patients age > 50 years or with postmenopausal status and was not limited to a certain type of breast cancer molecular classification [78]. Consistent with this work, we observed that Cox-2-controlled OAds were as effective at killing breast cancer cells as the Cox-2-positive A549 control cell line, indicating that breast cancer cells upregulate the Cox-2 promoter. Therefore, we thought that the Cox-2 promoter is an attractive candidate to control OAd replication in breast cancer patients. In previous studies, we established the effectiveness and specificity of the Cox-2 promoter in regulating OAd replication in laboratory settings (in vitro), in human tissue samples (ex vivo), and in vivo [18,53,54,79,80]. In our current investigation, we infected MCF-12A, a normal human breast epithelial cell line, with Cox-2 promoter-controlled vectors and observed a considerable decrease in replication compared to WT vectors, as evidenced by increased crystal violet staining and lower levels of Ad hexon protein expression. In contrast, both WT vectors and those controlled by the Cox-2 promoter exhibited robust replication and Ad hexon protein expression in breast cancer cell lines. Although replication of Cox-2 promoter vectors was not completely absent in normal breast cells, our data indicate that they replicate less efficiently in these cells compared to WT vectors, suggesting a greater preference for cancer cells. Additionally, our observations suggest that Cox-2-controlled replication vectors may undergo inefficient and incomplete replication in normal cells, as evidenced by the minimal expression of the hNIS transgene in noncarcinogenic MCF-12A mammary cells.

It is well-known that the incorporation of a TSP can often decrease the rate of oncolytic activity in cancer cells [18,80], and that is what we observed with crystal violet analysis of the Cox-2 promoter-controlled vectors in all tested cell lines. However, the oncolytic activity of Cox-2-controlled OAds remained strong even at low titers and was comparable to that of the OAd5 WT replication control. The Cox-2-controlled viruses were active in all tested cell lines and displayed improved selectivity for breast cancer cells. In addition, it would also be interesting to investigate the other promoters, especially those described to be mostly breast cancer tissue-specific, such as ErbB2 and MUC1, or a BCSC-specific promoter, like MDR, to further enhance the therapeutic potential of OAd expressing hNIS.

BCSCs are proposed to have heightened resistance to cancer therapies due to their relative quiescent state, enabling this population to evade standard-of-care treatments that target proliferating bulk tumor cells. We tested whether OAds could target such an undifferentiated population of cells with a reduced division rate. The use of Ad to effectively target cancer stem cells originating from cancer tissues other than breast was reported in chronic myeloid leukemia [81], glioblastoma [82], liver [83], lung [84], and gastric cancers [85]. However, little is known about the capacity of OAds to successfully target BCSC populations. Therefore, for this study, we employed a TaxR model that was previously characterized to express BCSC markers and to form tumorspheres in serum-free suspension environments [27].

Remarkably, in 2D monolayer cultures, all OAd5/3 vectors, especially those under the Cox-2 promoter, demonstrated superior efficacy in eliminating TaxR cells compared to control MCF-7 cells. Conversely, the Ad5 vector exhibited enhanced killing of parental MCF-7 cells compared to TaxR cells, as confirmed by crystal violet analysis. This heightened killing efficacy may stem from the elevated expression of Ad5/3 binding receptors (CD46 and/or DSG2) and decreased expression of the Ad5 binding receptor (CAR) in TaxR cells relative to parental MCF-7 cells. Nonetheless, further validation of this hypothesis is warranted in subsequent studies. hNIS gene expression (as determined by qPCR analysis) and protein levels (revealed through immunofluorescence staining analysis) were also found elevated in TaxR cells compared to control MCF-7 when using Cox-2-controlled vectors. This intriguing observation may potentially be linked to increased Cox-2 activity in the BCSC populations compared to other breast cancer cells. Although direct evidence is lacking, it is known that BCSCs exhibit heightened NF-kB pathway activity and prostaglandin expression [86]. Cox-2 expression is regulated by the NF-kB pathway, the effectors of which are often overexpressed in breast cancer cells [87], and Cox-2 in turn regulates prostaglandin levels, which play a crucial role in tumor growth regulation [88].

This is an important finding since, to our knowledge, there is only one publication reporting the use of TSPs in BCSCs. In this report, Bauerschmitz and others evaluated the potency of OAds controlled by four different TSPs (Cox-2, human telomerase reverse transcriptase (hTERT), multidrug-resistant (MDR) and alpha-lactalbumin (LALBA)) in killing BCSCs [72]. The authors concluded that high activity of the Cox-2 and MDR promoters in CD44^hi^/CD24^lo^ cells was responsible for high oncolytic efficacy of the viruses, which concords well with our findings in TaxR cells.

Once we demonstrated the active killing activity of our OAd5/3-NIS vectors in TaxR cells in 2D monolayer culture settings, we tested their potential to target BCSCs specifically. In previous work, Eriksson et al. demonstrated the potential of OAds (Ad5/3-Δ24 and Ad5.pk7-Δ24) to target isolated CD44^hi^/CD24^lo^ BCSCs from pleural effusions of breast cancer patients [89]. We were interested in whether our vectors could primarily reach the chemoresistant BCSC populations. To achieve this objective, we generated tumorspheres allowing for only stem-cell-like cells to grow into spheroids and infected them before or after the tumorsphere formation. We found that while our vectors exhibited comparable efficacy in killing tumorspheres to that observed in 2D monolayer cultures, it is noteworthy that a higher multiplicity of infection was required for the 3D culture, a common limitation of oncolytic viruses. Despite this difference in viral scale, the results underscore the potency of our vectors in targeting BCSCs across different culture settings. The oncolytic activity of all OAd5/3-based vectors was stronger in TaxR cells compared to parental MCF-7 cells. Impressively, the OAd5/3 Cox-2 ADP(−) hNIS vector was able not only to successfully inhibit tumorsphere formation upon initial infection but also to destroy already formed BCSC-enriched tumorspheres.

Furthermore, in our study, the most remarkable and surprising discovery was the ability of our vector to modify the behavior of paclitaxel-resistant ER+ BCSCs, rendering them responsive to paclitaxel once again when used in combination therapy. Specifically, in our cotreatment experiment, in contrast to MCF-7 cells, TaxR cells exhibited a reversal of chemoresistance when cotreated with the virus and paclitaxel. This resulted in a substantial killing effect of up to 60% upon virus infection. This is an unprecedented finding because, traditionally, chemoresistant ER+ BCSCs have posed a significant challenge in the field, often leading to treatment failures and disease progression. The ability of our vector to reverse resistance and enhance the efficacy of paclitaxel represents a breakthrough that has never been observed in this setting.

Overall, breast cancer treatment has undergone significant evolution in recent years with advances in surgical decision making, improved techniques for the delivery of radiation, and expanding options for systemic therapy including chemotherapeutics, targeted therapies, and immune checkpoint inhibitors. For those patients with advanced or metastatic disease, it is clear that combination regimens including some (if not all) of the aforementioned therapy components will be necessary to fight the cancer and to optimize survival outcomes. This strategy of multitargeted combination therapy is especially true for those patients who have demonstrated limited or no response to current lines of systemic therapy, as the treatment options are limited in these scenarios.

The current studies have demonstrated the remarkable potential of OAd-hNIS vectors to target and effectively eliminate human breast cancer cells, including the BCSC populations. The improved NIS expression in these cells could be a key factor in facilitating combination therapies with radioactive iodine. In addition, as demonstrated in recent clinical trials from four US cancer centers, ^18^F-fludeoxyglucose PET/CT could be effective in detecting regional and distant breast cancer metastases resulting in reducing false-positive imaging risk by half [90]. Thus, we believe that OAd-hNIS-based PET/CT imaging can potentially have important clinical application for breast cancer patients, as it will allow monitoring of undetectable BCSC micrometastatic deposits.

Furthermore, OAd5/3 Cox-2 ADP(−) hNIS has shown promising potential in treating chemoresistant BCSCs by demonstrating the ability to reverse paclitaxel resistance. While this discovery holds immense promise and opens up new avenues for the treatment of notoriously difficult-to-manage cancer subtypes, it is essential to approach these findings with caution, considering the in vitro preclinical context of our study. Additionally, the underlying mechanisms responsible for these remarkable effects remain elusive. Moving forward, it is imperative for the research community to focus on thoroughly investigating these mechanisms to fully understand and harness the potential of Ad-based clinical applications for chemoresistant BCSCs. These findings lay the groundwork for future studies that will delve deeper into the therapeutic potential of OAd-based vectors in vivo, which will be essential for their translation into clinical practice.

## Data Availability

The authors confirm that the data supporting the findings of this study are available within the article and its Supplementary Materials.

## Supplementary Materials

The following supporting information can be downloaded at: https://www.mdpi.com/article/10.3390/v16040567/s1, Figure S1: Adenovirus vector modifications for improved breast cancer cells infectivity; Table S1: Significance of Cox-2-controlled promoter killing ability compared to WT OAd.
