# Supplementary material for: Oncolytic Adenovirus for the Targeting of Paclitaxel-Resistant Breast Cancer Stem Cells
Source: Viruses. 2024 Apr 5;16(4):567. doi: 10.3390/v16040567 (PMC11054319; doi:10.3390/v16040567)
Supplement: Supplementary file 1 [file viruses-16-00567-s001.zip › viruses-2952170-supplementary.pdf]

viruses

MDPI

Supplementary Materials

Oncolytic Adenovirus for the Targeting of Paclitaxel-Resistant Breast Cancer Stem Cells

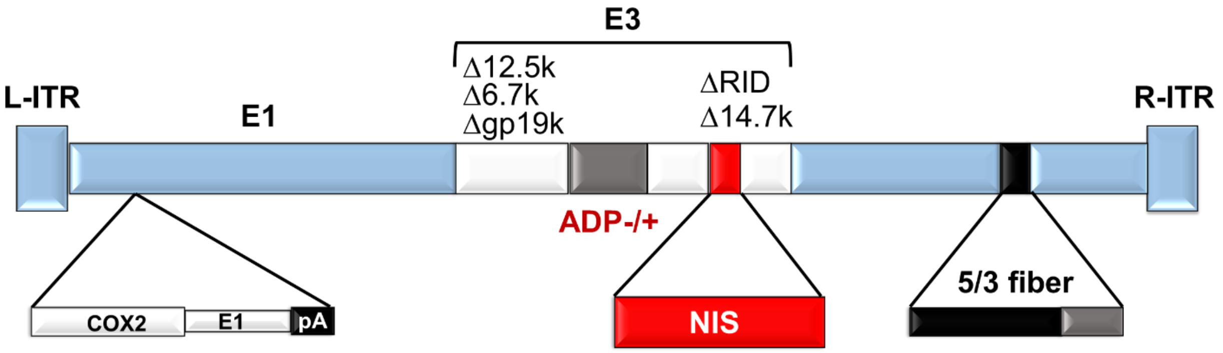

Figure S1. Adenovirus vector modifications for improved breast cancer cells infectivity. Constructs are based on adenovirus type 5 (Ad5) backbone and include deletion of the 12.5K, 6.7K, gp19K, RID-α, RID-β, and 14.7K genes from the E3 region. The Adenovirus Death Protein (ADP) gene was removed from “ADP(−)“ vectors set. The vectors are equipped with the Ad5/Ad3-modified fiber to overcome Coxsackie and Ad Receptor (CAR) deficiency and a Cox-2 promoter to restrict replication and gene expression to permissive tumors.

Table S1. Significance of Cox-2-controlled promoter killing ability compared to WT OAd. The statistical significance was determined by a Student’s t-test with a Mann-Whitney post hoc analysis. * $p < 0.05$. p.i.: post-infection.

Time p.i.	Titer (pfu/cell)	ADP Expression	A549	MCF-7	AU565	MDA-MB-468	MDA-MB-231
d3	10	+	* (WT>COX2)	* (WT>COX2)	* (WT>COX2)	* (WT>COX2)	* (WT>COX2)
		-	ns	ns	* (WT>COX2)	* (WT>COX2)	ns
	1	+	* (WT>COX2)	* (WT>COX2)	* (WT>COX2)	* (WT>COX2)	* (WT>COX2)
		-	ns	ns	* (WT>COX2)	* (WT>COX2)	ns
	0.1	+	ns	* (WT>COX2)	* (WT>COX2)	* (WT>COX2)	ns
		-	ns	ns	* (WT>COX2)	ns	* (WT>COX2)
d5	10	+	ns	* (WT>COX2)	ns	* (WT>COX2)	ns
		-	ns	* (WT>COX2)	ns	* (WT>COX2)	ns
	1	+	* (WT>COX2)	* (WT>COX2)	* (WT>COX2)	* (WT>COX2)	* (WT>COX2)
		-	ns	ns	* (WT>COX2)	* (WT>COX2)	* (WT>COX2)
	0.1	+	* (WT>COX2)	* (WT>COX2)	* (WT>COX2)	* (WT>COX2)	* (WT>COX2)
		-	ns	* (WT>COX2)	* (WT>COX2)	* (WT>COX2)	* (WT>COX2)
d7	10	+	ns	* (WT>COX2)	* (WT>COX2)	* (WT>COX2)	ns
		-	ns	* (WT>COX2)	ns	* (WT>COX2)	ns
	1	+	* (WT>COX2)	* (WT>COX2)	* (WT>COX2)	* (WT>COX2)	* (WT>COX2)
		-	ns	* (WT>COX2)	* (WT>COX2)	* (WT>COX2)	ns
	0.1	+	* (WT>COX2)	* (WT>COX2)	* (WT>COX2)	* (WT>COX2)	* (WT>COX2)
		-	* (WT>COX2)	* (WT>COX2)	* (WT>COX2)	* (WT>COX2)	* (WT>COX2)
